# Age at Initial Cleft Lip Repair Among Children in Al Madinah Region

**DOI:** 10.7759/cureus.49089

**Published:** 2023-11-20

**Authors:** Osman Suliman, Abdulaziz M Alraddadi, Faisal M Almutairi, Fadi A Almohammadi, Ziyad A Albakri

**Affiliations:** 1 Surgery, Al-Rayan Colleges, Al Madinah, SAU; 2 Medicine and Surgery, Al-Rayan Colleges, Al Madinah, SAU

**Keywords:** complication, rabbit lip, plastic surgery, hairlip, cleft repair, cleft lip

## Abstract

Background: Cleft lip and palate consists of a wide spectrum of anomalies affecting the oral cavity and lips and can have enduring adverse effects on health. The age at which surgical interventions are done is crucial as it can influence the outcomes. This study aimed to determine the age at which initial cleft lip repairs were performed and the proportion of individuals who underwent additional surgeries to address post-repair complications.

Methods: A cross-sectional study involving 120 participants was conducted. Data was collected by distributing an online questionnaire to parents of children who had undergone initial cleft lip repair in the Al Madinah region.

Results: The study found that the average age for the initial cleft lip repair in Medina is 0.46 ± 0.27 years. Post-repair complications were observed, including hearing loss in 10.9% of children, heavy breathing issues in 32.8%, an imbalance in facial expression in 47.9%, and swallowing problems in 3.4% of children even after surgery. Approximately 40% of parents sought further procedures to mitigate complications.

Conclusion: The study indicated that the first cleft lip repair in Medina typically occurs during the first year of life. However, more extensive research is needed to assess the efficacy of procedures within the city. Further studies should be conducted to provide a more comprehensive understanding of these conditions and the outcomes of their treatments.

## Introduction

A cleft lip and palate (CL/P) is a congenital craniofacial anomaly characterized by a split in the roof of the mouth caused by tissue that does not fuse during development, leading to a readily noticeable deformity in newborns [1,2]. The etiology of CL/P is complex and multifaceted, influenced by both genetic and environmental factors. Orofacial clefting can manifest as a non-syndromic condition, occurring in about 85% of cases of cleft lip with or without cleft palate and roughly 45% of cases of cleft palate alone [3]. With the adoption of the Sustainable Development Goals and the passing of World Health Assembly resolution 68/15, titled "Strengthening Emergency and Essential Surgical Care and Anesthesia as a Component of Universal Health Coverage [4]," member states have committed to expanding the provision of essential surgery to achieve universal health coverage within their respective nations. It has been suggested that by enhancing accessibility to pediatric surgical care, we can significantly reduce childhood mortality and lifelong impairment, especially in low- and middle-income countries (LMICs) [5]. CL/P is among the most common craniofacial birth conditions, affecting approximately one in every 700 live infants [1], and is more prevalent among male children [6].

Studies conducted in Saudi Arabia showed that cleft palate (CP) was the most common type of orofacial cleft, followed by cleft lip and unilateral cleft lip and palate [7]. Moreover, 16 out of 24,367 live births were diagnosed with CL/P, resulting in a total frequency of 0.65/1000 live births [8]. The diagnosis of cleft lip (CL), with or without an associated CP, does not require specialized knowledge or testing. Therefore, the importance of this condition as an indicator for pediatric surgery remains unaffected by diagnostic constraints and delays. Surgical procedures for repairing CL are typically performed between three to six months of age. The timing of surgical repair and the accompanying speech and language therapy significantly affect the acute and long-term well-being of CL/P patients. Over the past few decades, CL/P treatment protocols have evolved significantly alongside the growth of evidence-based medicine. In the late 1970s, palate repair was recommended at around 24 months of age, while by 2000, it was suggested to be performed closer to 12 months [9]. Some studies suggest that the optimal age for cleft lip surgery is typically between 3 to 5 months of age. However, a study retrospectively assessing the outcomes of surgeries performed on two groups: one group, which underwent the surgical procedure between 3 and 7 months of age, and the other group, which had surgery after seven months, showed that both groups achieved complete closure of the hard and soft palates by 13 months, with an average age of 7.5 months [10]. Some studies reported successful operations at the mean age of 34.8 days (13-69 days) [11], while others were done at 12 years of age with normal speech development later in life [1,6,12].

With the lack of consensus on how to repair CL/P, there is a need to research the optimal age of CL/P repair to understand the ages at which repair leads to more or less complications. Therefore, this study aimed to determine the age at which initial cleft lip repair is performed in Saudi Arabia and the proportion of children who experienced post-repair complications needing additional surgical procedures.

## Materials and methods

Study design and population

A cross-sectional study collected data through an online questionnaire in the Al Madinah Al Munawwarah region from July to October 2023. The study targeted children born with CL who had undergone initial repair, whether unilateral or bilateral or associated with cleft palate in the Al Madinah Al Munawwarah region. Children not born with cleft lip, even if they had other congenital disabilities within the region or resided outside Al Madinah Al Munawwarah, were excluded. With a population of 2.13 million in the Al Madinah Al Munawwarah region and an estimated incidence of CL/P at 0.65 per 1000, there were approximately 1386 individuals with CL/P. Out of these, only 12.5% were observed to have cleft lips, resulting in a population of 173 individuals in Medina. Considering a 95% confidence interval, the sample size for this study was determined to be 120 using Raosoft, and we used a convenience sampling technique to select the eligible participants due to its simplicity.

Data collection tool and procedure

An online questionnaire was used to collect data from the parents of children. The questionnaire collected demographic data of the children, data on the relationships among parents, the type of oral cleft, the type of oral hole, the surgical procedures undertaken, and any complications following the procedures. Data on the age at which the first repair was done were collected from the hospital patient's files.

Data analysis

Data obtained from the study was analyzed using the Statistical Package for Social Studies Program (SPSS, V. 21.0. IBM: Chicago). We performed descriptive statistics and presented categorical data in frequency (%), and continuous data in mean ± standard deviation (SD).

Ethical considerations

Ethical approval for the study was obtained from the Al-Rayan Research Ethics Committee, registered with the National Bioethics Committee in KACST, Saudi Arabia (Ref No: HA-03-M-122-051). Online consent was secured from the parents before completing the online questionnaire. All personal data was handled with strict confidentiality.

## Results

**Figure 1:**
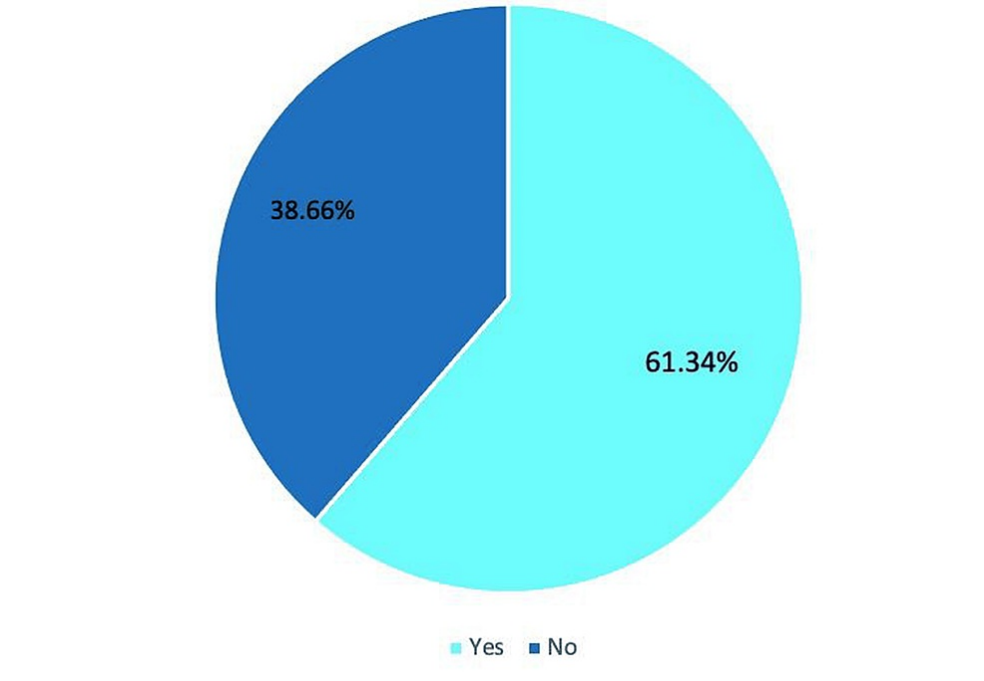
The family relation among parents The data has been represented as a percentage occurrence of the variable

We included 120 participants, and the mean current age of the participants was 6.98±6.05 years. The mean age at the first procedure among Saudi children was 5.52±3.24 months. Most participants (56.3%) were males, while 43.7% were females. Participants' characteristics are shown in Table 1. We found that 38.66% of parents were somehow related to one another before marriage (Figure 1).

**Table 1:** Demographic characteristics of participants The data has been represented as the frequency and percentage of occurrence of the variables; SD: Standard deviation; %: Frequency

Demographic characteristics	
Age	Mean±SD
Present Age years	6.98 ± 6.05
Age at the time of repair years	0.46 ± 0.27
Gender	Frequency (%)
Male	67 (55.8%)
Female (frequency %)	53 (44.2%)

**Figure 2:**
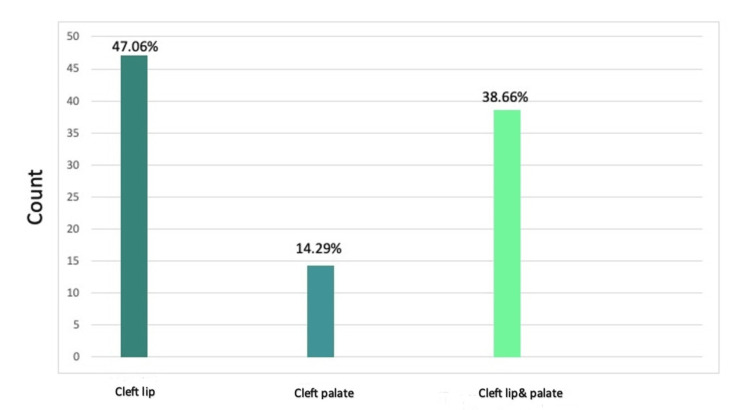
The type of cleft lip The data has been represented as a percentage occurrence of the variable

**Figure 3:**
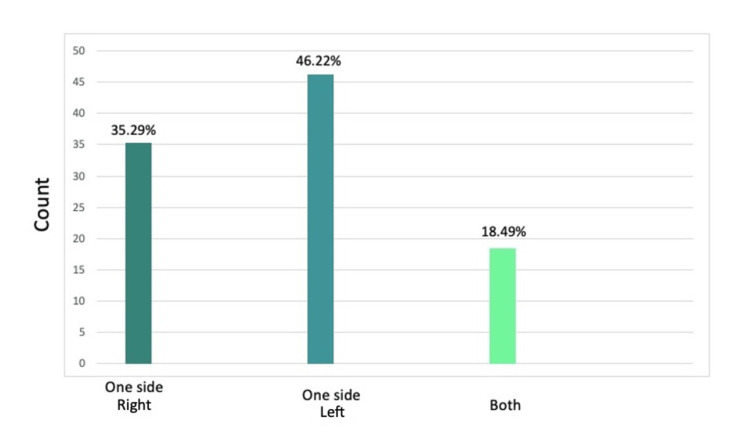
Type of oral hole in cleft lip The data has been represented as a percentage occurrence of the variable

Most children (47.06%) had a cleft lip, 14.29% had a cleft palate, and 38.66% had a cleft lip and palate (Figure 2). Moreover, most children (46.22%) had left-side holes, 35.29% had right-side holes, and 18.49% reported having both-side holes (Figure 3).

Only almost a quarter (26.6%) of parents had education on cleft lip and its recommended surgeries. The findings showed that 13.4% of the children had additional congenital disabilities. Notably, 92% of the children received treatment and procedures for CL/P. However, only 39.2% of the participants underwent a secondary operation for Rabbit's lip. Approximately 60% of the participants had their palates repaired, while the throat roof was surgically adjusted in 40% of the children. Furthermore, bone vaccination was administered to 60.8% of the participants (Table 2).

**Table 2:** Procedures performed to treat cleft lip The data has been represented as the frequency and percentage of occurrence of the variables; %: Percentage

Variables	Yes, frequency (%)	No, frequency (%)
Family education on Cleft lip	32 (26.6%)	88 (73.4%)
Any Other congenital disability	16 (13.4%)	104 (86.6%)
Was the Rabbit lip or throat roof operation?	110 (92.4%)	10 (8.3%)
Was the rabbit lip or a cleft palate treated	110 (91.6%)	10 (8.3%)
Was another operation to be performed for Rabbit lip?	47 (39.2%)	73 (60.8%)
Was a process made for the Rabbit lip and nose	47 (39.2%)	73 (60.8%)
Has the roof of the throat been fixed?	72 (60%)	48 (40%)
Was there another operation for the cleft palate?	48 (40%)	72 (60%)
Was a bone vaccination made?	73 (60.8%)	47 (39.2%)

Parents were asked if the children experienced any complications after surgery. As shown in Table 3, 41.7% of the children had concealed teeth, while another 41.7% encountered speech difficulties. A smaller percentage (12.5%) reported some degree of hearing loss. Additionally, 33.3% experienced issues related to excessive breathing, 47.5% faced imbalances in their expression and audio, and the majority (76.7%) had difficulties swallowing before the procedure. However, these issues persisted in only 3.4% of the children after the procedure.

**Table 3:** The complications that arise after the procedure The data has been represented as the frequency and percentage of occurrence of the variables; %: Percentage

Variables	Yes, frequency (%)	No, frequency (%)
Have the teeth hidden?	50 (41.7%)	70 (58.3%)
Are there problems in communication?	50 (41.7%)	70 (58.3%)
Is there a loss of hearing	15 (12.5%)	105 (87.5%)
Is there a breathing excessive	40 (33.3%)	80 (66.7%)
Is there an imbalance in expression or an audio disturbance?	57 (47.5%)	63 (52.5%)
Is there difficulty swallowing before the reform	92 (76.7%)	28 (23.3%)
Is there difficulty swallowing after reform?	4 (3.4%)	116 (96.6%)

**Figure 4:**
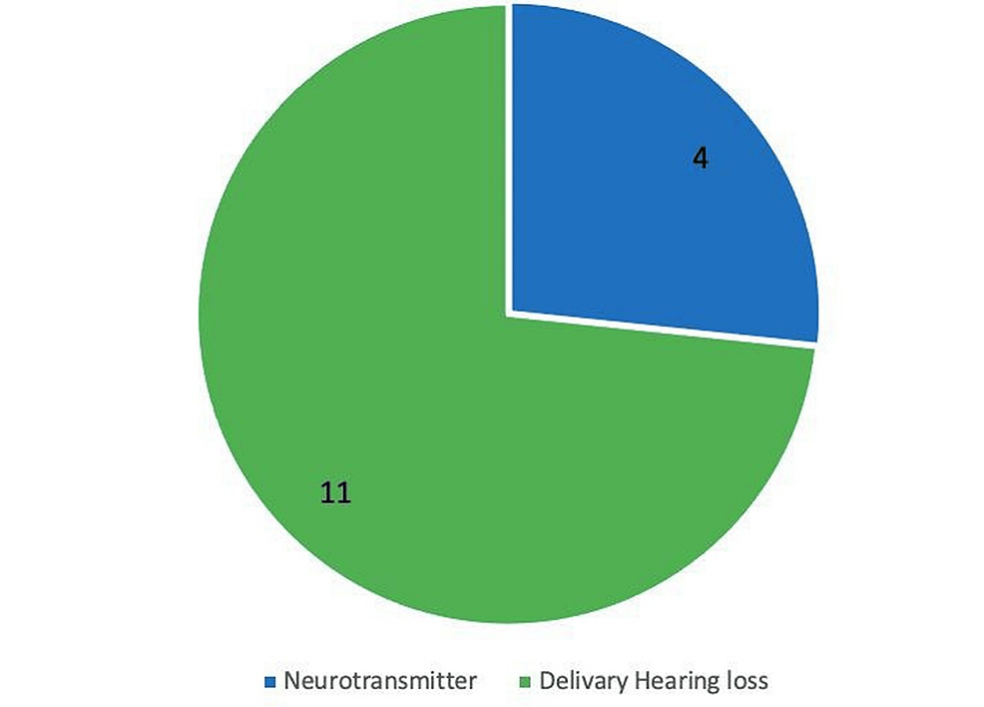
Type of Hearing loss after procedure

Of the 15 participants who were recorded to have some hearing loss, 4 participants had hearing loss from neurotransmitters, and 11 had the delivery hearing loss issue (Figure 4).

## Discussion

This study aimed to investigate the average age at which the first repair for CL/P is performed in Saudi Arabia, identify the complications children experience following these repairs, and determine the proportions of patients needing additional surgeries to address the complications. By exploring the prevalence of post-repair complications necessitating additional surgical interventions, the findings will inform evidence-based protocols for cleft lip and palate treatment and refine surgical practices related to craniofacial anomalies, ultimately enhancing the quality of care. Understanding the optimal age for cleft lip repair and assessing associated complications will help inform healthcare policies and reduce the burden of lifelong impairment associated with CL/P. The findings might also influence healthcare decision-making toward optimal health outcomes for children born with cleft lip and palate.

The findings of this study revealed that the mean age at which children underwent their first repair was 0.46 ± 0.27 years. Cleft lip and palate are often of concern to parents due to their aesthetic impact, and it is recommended that repairs be performed before the child's first year of life. Previous studies have shown better results when repairs are conducted within the first year of life [13,14]. A study in Nigeria reported that 75% of participants underwent repairs before their first birthday [15]. 

A previous meta-analysis showed that the prevalence of cleft lip was 0.3 in every 1000 live births (95% CI: 0.26-0.34), while the prevalence of CL/P was 0.45 per 1000 live births (95% CI: 0.38-0.52) [16]. In terms of the type of cleft lip, a study in the United States of America found that unilateral left lip cleft was the most common [17]. Our study aligns with the previous studies and shows that the most prevalent type was unilateral right-sided cleft lip (46.2%), followed by unilateral left cleft lip (35.3%), and then bilateral cleft lip (18.5%).

It is well known that there is a relationship between consanguinity and CL/P and other congenital abnormalities [18], which may have increased the risk of CL/P for the children in our study since we found that the parents of 38.66% of children were somehow related. Consanguineous marriages, particularly between first and second cousins, are prevalent in Saudi Arabia and deeply rooted in the country's traditions. An investigation involving 3212 Saudi families revealed that 57.7% of the families were in consanguineous marriages, with the highest rate at 86.6% in Samtah and the lowest at 34.3% in Abha, both in the southern part of the country [19]. However, there is a need for more comprehensive research on consanguinity in Saudi Arabia.

Regarding the general treatment approach, surgical teams typically initiate consultations with parents of babies diagnosed with CL/P during pregnancy or at delivery, and treatment continues into adulthood [20]. Over time, families and patients develop a unique bond with their pediatric cleft team specialists, making the transition to clinicians who primarily treat adults bittersweet [17]. In our study, only 26.1% of parents reported receiving education on cleft lip, indicating poor management services for CL/P in the Al Madinah Al Munawwarah region. This highlights the need for measures and strategies involving healthcare providers and parents to raise awareness and education about CL/P holistic management. The findings also revealed that 92% of children had undergone Rabbit's lip repair. However, only 39% opted for the operation, and 40% underwent a throat roof-fixing procedure. This indicates intervention variations, aligning with other studies. A study in the United Kingdom examined surgical interventions and treatments performed over twenty years. It found variations in the timing and pattern of interventions performed by practitioners, with only 38% of practitioners consistently adhering to one treatment pattern for their patients [21].

Regarding complications, most children experience speech and hearing difficulties, as documented in other studies [11,22,23]. The complications most result from possible nerve, blood vessel, muscle, and auditory canal injuries, which are mostly temporary. Damage to nerves involves the lingual and inferior alveolar nerves (IAN) [24]. Surgical side effects may include bleeding, edema, discomfort, infection, and delayed or nonunion [11,22]. These side effects are usually temporary, resolving over time. This study, however, focused on the long-term impacts of cleft lip, even after procedures, focusing on complications related to primary senses. Given the limited availability of data on long-term complications, further studies should be planned to investigate the long-term effects of cleft lip and surgical interventions on the quality of life.

## Conclusions

This study revealed that the average age at which the initial repair procedure was performed was 0.46 ± 0.27 years, aligning with available literature evidence. Complications, such as hearing loss, imbalances in facial expression, and excessive breathing issues, were observed post-repair interventions. Notably, approximately 40% of parents opted for additional procedures to address these complications, indicating their contributions to parental concerns and impact on children's quality of life. We recommend further comprehensive studies examining the interplay of various factors in this context and the challenges children face after cleft lip repair, focusing on formulating effective policies to minimize the need for additional surgeries.
